# A discursive exploration of public perspectives on placebos and their effects

**DOI:** 10.1177/2055102919832313

**Published:** 2019-02-15

**Authors:** Doug I Hardman, Adam WA Geraghty, Jeremy Howick, Nia Roberts, Felicity L Bishop

**Affiliations:** 1 University of Southampton, UK; 2 University of Oxford, UK

**Keywords:** communication, critical health psychology, illness perception, internet, placebo

## Abstract

There is increasing evidence that placebos could be effective in clinical practice. However, knowledge of public perspectives on placebos is underdeveloped. We conducted a discourse analysis of internet comments on news articles related to placebos, aiming to improve this knowledge for clinicians and researchers. We developed two discursive constructs of the placebo. The dominant construct of the ‘placebo pill’ informs a paradoxical understanding of placebos that closes down treatment. The less-prevalent counter-discursive construct of the ‘treatment process’ frames placebos as potentially viable within modern evidence-based medicine. We discuss the opportunities and challenges of this alternative understanding of placebos.

## Background

Within medical research, a ‘placebo’ is commonly understood as an object used in randomised controlled trials to control for, among other factors, the psychological, social and cultural effects of treatment. Our focus is on the clinical use of placebo treatment, understood broadly as when something like these factors are deliberately exploited by healthcare professionals during treatment, outside the context of a trial. The clinical placebo has a long history (Shapiro and Shapiro, 1997), but current understanding has developed from the early notion of an ‘ineffective’ treatment used to please patients, to one of an active agent in its own right (Kaptchuk, 1998; Kaptchuk and Miller, 2015; Kerr et al., 2008; Wolff et al., [1946] 2013). In this study, we discursively explore public perspectives on clinical placebos and their effects.

There is increasing evidence that placebo treatment may have clinical utility (Benedetti, 2014; Evers et al., 2018; Vase et al., 2002; Wampold et al., 2005). For example, treating pain by inducing the production of endogenous opiates (Benedetti, 1996; Levine et al., 1978) and treating Parkinson’s disease by inducing the release of endogenous dopamine (De la Fuente-Fernández et al., 2001). Moreover, healthcare professionals are known to use placebo treatment in clinical practice for different reasons such as to improve care, manage patients’ expectations and cope with uncertainty (Bishop et al., 2014b; Comaroff, 1976; Tilburt et al., 2008).

However, although placebo treatment is common, its frequency of use and healthcare professionals’ attitudes towards placebo treatment vary significantly (Fässler et al., 2010; Linde et al., 2018). Furthermore, despite potential clinical utility, there are considerable definitional and ethical disagreements surrounding placebos and their effects (Alfano, 2015; Blease, 2011; Miller and Brody, 2011; Miller and Colloca, 2009). For example, placebo effects have been conceived as follows: the psychological effects of ‘inert’ substances (Beecher, 1955); the psychosocial context of treatment (Colloca and Miller, 2011); the effects of healing rituals and symbols (Brown, 2013; Kaptchuk and Miller, 2015; Miller and Colloca, 2010) and the response to enculturated meaning (Moerman, 2002). At present, some promising attempts at a synthesis notwithstanding (e.g. Howick, 2017; Ongaro and Ward, 2017), an integrative theory of placebos does not yet exist (Miller et al., 2009).

Compounding this lack of consensus, the increasingly nuanced understanding of placebos and their effects in the research community is becoming progressively detached from public perspectives. This is especially significant for a process that, as noted above, is increasingly understood as entangled with meaning and social interaction (Moerman, 2002; Moerman and Jonas, 2002). If, as seems reasonable, placebo effects cannot be explained by the effects of an ‘inert’ substance, then the focus should shift towards understanding how patients interpret something like this phenomenon (Moerman, 2013).

The definitional and ethical disagreements around placebos and their effects are evident in public perspectives. For example, some patients take a consequentialist view, stating that placebos are acceptable as long as it helps. Others state that placebos are unethical because they, supposedly, require deception, violating a deontological commitment to patient autonomy (Bishop et al., 2014a; Feffer et al., 2016). In addition, although there is an increasing focus on patients’ perspectives (Hull et al., 2013; Lynoe et al., 1993; Tandjung et al., 2014), research in this area is underdeveloped.

Given the ambiguity in placebo studies research, particularly regarding the views of healthcare professionals, researchers conducted a study exploring placebo use by general practitioners (GPs) in the United Kingdom (Howick et al., 2013). This included findings that 77 percent of surveyed GPs used placebos – defined very widely – at least once a week and that most respondents believed placebos – again defined very widely – to be ethical in some circumstances. These findings attracted significant media attention from major online news organisations.

Perhaps due to the contentious nature of the debate, the news articles resulted in many readers commenting on the respective websites. These comments give an indication of public perspectives on placebos and their effects; commentators were largely responding to the findings of the study, interpreted by journalists, or to the placebo phenomenon more generally. We conducted a discursive exploration of these comments, aiming to understand (1) how members of the public conceptualise placebos and their effects; (2) how the ethical status of placebo use is publicly negotiated and (3) how these conceptualisations and ethical negotiations might open up and close down potential modes of clinical practice.

## Method

### The data

Data were collected from internet comments on six UK news articles responding to Howick et al.’s (2013) study on placebo use by UK GPs. The included news organisations reflect a political cross-section of the mainstream UK media. Howick et al.’s study was published in March 2013, as were the news articles. Data collection was restricted to UK organisations to set the conditions for deeper socio-cultural analysis.

The data consist 930 comments, over six sources, covering 55,410 words. The typical length of each comment is between 40 and 70 words. One data source dominated, with 445 comments. The other five sources have 202, 176, 63, 43 and 1 comment, respectively.

The data are located on popular, open-access online news sites; therefore, it is reasonable to assume that the data are already public. However, in the context of internet-mediated research, the distinction between private and public space is complex (British Psychological Society, 2013). Although the data can reasonably be considered public, commentators did not contribute their opinions with the explicit understanding that they would be used for research or disseminated in a different context. Therefore, the specific news articles are not named in this study, and all pseudonyms used by online commentators are anonymised to better protect their confidentiality: this is also stipulated in the ethical clearance given for this study. In the analysis, each data source is identified by a letter (A, B, C, D, E, F). Within each data source, commentators are then sequentially numbered (A1, A2, A3,…, etc.).

### Mode of analysis

Discourse analysis can be understood broadly as a method of analysing the content, structure and performance of language. In psychology, it is broadly split into two approaches: ‘discursive psychology’ is concerned with discourse practices, what people do with language; and ‘critical discourse analysis’ (CDA) concentrates on the discursive resources supporting language, what sort of being-in-the-world is available to people (Willig, 2013). Given the contention surrounding placebos and their effects and the importance of socio-cultural factors in their comprehension, we used CDA, as it allowed us to analyse the structures of meaning behind the placebo phenomenon and how people utilise the phenomenon in practice.

There are a range of methodological approaches termed CDA (Fairclough, 2015; Parker, 2015; Wodak, 2007). Our approach was orientated towards exploring social structures and systems of meaning (Parker, 2015); our analysis was grounded in the principles of ‘an attention to history, theory and subjectivity’ (Parker, 2015: 18). However, unlike more prevalent forms of CDA, our approach was mediated by a pragmatist approach to inquiry and a more neutral interpretation of power relations (Dewey, 1958; Rorty, 1982; West, 1999).

We adopted a two-phase, abductive analytic approach outlined by Potter and Wetherell (2010): identify patterns in the data and then identify functions and consequences. We first identified the predominant constructs of the discursive object in question (the placebo) through a close reading of all comments. We then identified the controlling metaphors, notions, categories and norms that support or suppress these constructions – the discursive resources – again through a close reading of the comments. Next, we explored the variations, relationships and tensions between discursive resources, including how they are produced and promoted. We did this by comparing and contrasting comments across the data sources and also by analysing interactions between commentators within each data source. We then situated the discursive resources and relationships between resources within wider ways of being-in-the-world: discourses.

After identifying patterns in the data, we focussed on the functions of commentators using certain discursive resources and identified the subject positions they took within discourses. Finally, we identified the consequences of commentators taking various subject positions within discourses and how this influences the intelligibility, availability and legitimacy of the discursive constructs. Throughout the analytic process, the integration of theory was not an explicit phase but incorporated throughout the analysis (Wodak, 2007).

## Analysis

We developed two discursive constructs of the ‘placebo’: the dominant discursive construct of the ‘placebo pill’ and the less-prevalent counter-discursive construct of the ‘treatment process’. We use these two constructs to frame the analysis.

### The placebo pill

In his interpretation of the consequences of modernity, Anthony Giddens (1990: 21) identified the disembedding of social systems, whereby social relations are ‘[removed] from local contexts of interaction’ and restructured. One of the mechanisms he identified as intrinsic to this process is the creation of symbolic tokens, used ‘without regard to the specific characteristics of individuals or groups that handle them at any particular juncture’ (Giddens, 1990: 22). A common example is money, but our dominant discursive construct is a narrower example of such a symbolic token: the placebo pill.

Commentators in our study commonly conceived of a placebo as an ‘inert’ substance that is given to a patient. This common lay definition is similar to that provided by Henry Beecher in his famous (1955: 1602) paper, where placebos are ‘pharmacologically inert substances … having a psychological effect’. However, as other researchers have noted, this definition is problematic, as a ‘placebo’ cannot be understood coherently without reference to the patient, condition and therapeutic theory in question (Grünbaum, 1986; Howick, 2017). Moreover, nothing is actually inert – one can treat any substance in physico-chemical terms if one chooses to do so – and describing a placebo in this way is, therefore, paradoxical. For example, ‘even the proverbial sugar or bread pill will prove far from inert in patients with insulin dependent diabetes or with gluten intolerance, respectively’ (Howick, 2017: 1365). Despite these issues, the reification of the placebo pill as a disembedded symbolic token dominated discussion. We identified three discursive resources supporting this construct: market exchange, individual decision-making, and biomedicine.

### Market exchange

Market exchange emerged as a central resource that was used to support the dominant construct of the placebo pill. By conceiving of a placebo as an object that is given by a healthcare professional to a patient, the norm of market exchange is made available to guide human action. This was common across the data sources:

Data source A, Commentator 4 (A4): This is fraud. The patient is told that he/she is getting a certain drug and then goes and buys this at the chemists. But the patient is in fact paying good money for what is effectively water. It is considered fraud even to sell something that poses even as any illegal drugs! Why should the law be any different for legal drugs especially. Unbelievable, there is no hope in this country!

F2: Are you really saying that some doctors give patients prescriptions that the patients then take to Boots to hand over some of their hard-earned cash for a packet containing nothing but little lumps of sugar? If that is so, and it is done on a large scale, this practice should be exposed and the perpetrators prosecuted.

E5: So let me get this right. Supermarkets are vilified and a national crisis created when suppliers are found to have included horsemeat, which is harmless, in what are sold as beef products. But if some crew of ‘doctors’, who are too greedy to tell people that they don’t need treatment/pills and too lazy to explain why give out placebos ie fake treatments that deceive the public, that’s fine.

Drawing on an individualist discourse, commentators focussed on themselves as consumers within a market, whereby the aim was to ensure they were seen to get a fair deal from their healthcare professional. A common refrain was that ‘I’ll pay for my prescription with placebo money then’ (E4) or that ‘placebos are fine, as long as the chemist will accept a dud cheque for the prescription charge’ (E1). To this end, commentators promoted the primacy of contractual relations:

A5: If I found out I’d been given a ‘placebo’ I would be furious at the waste of my money on the prescription.

F1: Hang on a minute! Prescribing placebos is surely a conspiracy to commit fraud? After all we pay chemists £7.65, £7.85 from April, per prescription. Bloody expensive Smarties!

Given the existence of prescription charges in England, if a placebo is understood as a pill, some commentators may reasonably feel they are involved in a market exchange, rather than in the promoted institutional framework of free-at-the-point-of-use healthcare. Moreover, if as the geographer David Harvey (2005: 42) posited, society is dominated by a ‘market-based populist culture of differentiated consumerism and individual libertarianism’, it is not surprising that this appeared to inform commentators’ understanding of placebo treatment. However, a common criticism is that such an individualist society negatively affects social relations, promoting division (Giddens, 1990; Habermas, 1984). One consequence of this is the second discursive resource supporting the placebo pill construct: individual decision-making.

### Individual decision-making

Commentators often promoted a pejorative notion of the placebo, setting placebo treatment against that used for patients with ‘genuine’ illnesses.

A12: Probably this article refers to those who insist on being given something instead of sucking it up for a couple of days. Instead they can suck on a placebo.

A15: All that this report does is confirm my deeply held suspicions that many of the people who sit in the doctors’ waiting room have absolutely nothing wrong with them other than anxiety or a need for attention.

Within such an individualist discourse, healthcare was presented as a scarce resource by which productive work can be maintained: ‘We all know someone who goes to their GP/ hospital at every given chance. What annoys me is that these time wasters waste a huge amount of resources’ (A16). Informed by this, some commentators resisted control by the healthcare system, taking the position of self-reliant patients making individual medical decisions.

E9: I have rarely ever taken something prescribed by my GP. On the occassions [sic] that I have, I have googled the drug and checked the dosage before taking it.

A30: I haven’t taken a medicine I didn’t research since I was 12. If you’re being given a treatment that is mis-labelled chemically, that’s a crime, if you’re not researching what treatment you’re taking, thats your fault.

If the placebo pill, as a symbolic token, can be conceived of as a disembedding mechanism, then this self-reliance can be understood as resistance to another such mechanism – expert systems. For Giddens (1990: 28) ‘expert systems are disembedding mechanisms because, in common with symbolic tokens, they remove social relations from the immediacies of context’. Some commentators undermined the expert system of healthcare and the social position of doctors, noting that ‘in any case, most GPs remain clueless about the finer details of pharmacology’ (D2) and that ‘doctors are all too ready to give out antibiotics like sweeties … I always refuse them and the doctor gets quite confused basically says go home and get over it’ (E11). There is tension between, on one hand, embracing the symbolic token of the placebo pill, and on the other hand, the lack of faith in systems beyond one’s full comprehension.

However, although some commentators took self-reliant positions, others took positions as passive patients, reinforcing faith in expert systems:

A29: Even with the aid of the internet it is highly arrogant to believe you know better than a doctor who spent years training to be what they are.

E1: I don’t care what I’m prescribed as long as a) it works and b) doesn’t cause me any additional damage. Nothing wrong with a placebo if it does the job!

F6: I don’t care whether it’s a placebo or not, I just want to feel better and if tricking my mind is part of that, so be it.

Explicitly giving control to the healthcare profession in this way, patients ceded their role in the decision-making process.

Within the placebo pill construct, therefore, patients took active (resisting expertise) and passive (ceding control) individual decision-making positions. Both these approaches were informed by the third discursive resource supporting the placebo pill construct: biomedicine.

### Biomedicine

It was common for commentators to focus on physical explanations at the expense of social, cultural or intersubjective factors. This was manifest through a number of techniques. First, through bifurcating nature into different degrees of reality, for example stating that ‘a placebo is NOT a drug, it is an inert substitute which is known to have zero direct physical effect’ (A24) or that ‘there is a plausible (though as yet unproven) theory that the placebo effect has a *real* physiological effect by stimulating the body’s immune system to kick fully into swing’ (A26).

Second, despite the often broad effects of pharmacological treatment, some commentators set placebo pills against what they viewed as proper, ‘targeted’ drugs, for example, noting that ‘when having to pay out for a prescription, you should have a drug that is designed for your problem’ (A7).

Third, commentators positioned placebos as ineffective against ‘genuine’ illnesses, viewing them merely as something to placate a patient. For example, noting that ‘the placebo would not work if the illness was genuine and the patient would then have to go back’ (E6) and that ‘if someone had a genuine illness and was prescribed a placebo, it would become obvious very quickly that the tablet wasn’t working, and they would have to go back to the doctor’ (E7).

It is not surprising that biomedicine was a dominant discursive resource. After all, to extend Quine’s (1953: 44) analysis to medicine, ‘the myth of physical objects is epistemologically superior to most in that it has proved more efficacious than other myths as a device for working a manageable structure into the flux of experience’. Medicine has been historically successful precisely because it has adopted the myth of physical objects. However, such a myth has its limits. The paradox inherent in solely conceiving of placebos biomedically, as pills, perhaps reflects the efficacious limits of biomedicine’s myth of physical objects. This has implications for medical practice, particularly in primary care, where clinicians are expected to practise person-centred medicine, synthesising technical medical knowledge with individual patient values.

### The treatment process

Although the placebo pill construct dominated, from the data we developed a less-prevalent counter-discursive construct – the treatment process. Through this construct, the placebo is not conceived of as a substance, such as a pill, but as the whole clinical treatment process in which a material substance may or may not be involved. We identified two discursive resources supporting this construct: self-healing and shared decision-making.

#### Self-healing

The first discursive resource is self-healing. In contrast to the notion of the passive patient, prevalent in the placebo pill construct, commentators presented themselves as active actors in the treatment process, focussing on their own ‘self-healing’ capacity:

D4: It is my knowledge that the human body has an amazing/powerful way of self healing. You will need some in depth education in understanding the amazing way in which the human body defends itself from viruses, bacteria and many other illnesses. The human body is very efficient at resolving it’s own health issues without medical intervention or drugs

A41: The body has a remarkable ability to heal itself. What placebo’s [sic] show is that we just need to convince ourselves that we will be better and in many cases the body does the rest. This is the root I think of all miracle cures. To poo poo placebo’s [sic] and other alternative treatments is to shut the door on allowing the body to heal itself.

Moreover, some commentators explicitly stated their views on the limitation of the biomedical model and its delivery within the institution of England’s National Health Service (NHS):

C6: But the reality is that many doctors, and the NHS structure itself, is such that these methods are not treated as important enough to prioritise because their methodologies do extend beyond the mechanical, they extend into psychological wellbeing, breathing, state of mind, awareness, none of which is recognised by the popular interpretation of a biomechanical medical model.

A35: the body is a fantastic piece of kit with great healing abilities. Perhaps education on this is better than perpetuating the myth that the medical model has all the answers, especially when it doesn’t, hence the article.

Others noted that ‘the body is perfectly capable of producing its own drugs and placebo may work by illiciting [sic] this response’ (F8), echoing the numerous studies which have shown that placebo treatment can, for example, stimulate the production of endogenous opiates (Amanzio and Benedetti, 1999; Eippert et al., 2009; Gracely et al., 1983; Pecina and Zubieta, 2018).

By accepting the limitations and fallibility of biomedical knowledge, commentators did not present themselves as active agents set against treatment – as with the self-reliant patient – but as patients taking an active role in treatment, for example, noting that ‘in many cases placebo’s [sic] work by *enhancing* your bodies healing mechanisms’ (A34). This broadly concurs with recent embodied and enactive accounts of placebo effects (Ongaro and Ward, 2017; Thompson et al., 2009), whereby ‘the living body is a self-producing and self-maintaining system that enacts or brings forth relevance, and that cognitive processes belong to the relational domain of the living body coupled to its environment’ (Varela et al., [1991] 2016: xxv). Although there are a range of broadly enactive accounts (Hutto and Myin, 2012; Thompson, 2010; Varela et al., [1991] 2016), it is this notion of an active, autonomous organism co-dependent with its environment that characterised our findings. This leads to the second discursive resource: shared decision-making.

### Shared decision-making

Developing his interpretation of the consequences of modernity, Giddens (1990: 79) noted that ‘people [increasingly] live in circumstances in which disembedded institutions, linking local practices with globalised social relations, organise major aspects of day-to-day life’. However, he complemented the notion of disembedding with *re-embedding*, meaning ‘the reappropriation or recasting of disembedded social relations as to pin them down (however partially or transitorily) to local conditions of time and place’ (Giddens, 1990: 79–80).

For Giddens, the disembedding mechanisms (symbolic tokens or expert systems, understood together as abstract systems) interact with re-embedded contexts of action. One of these contexts is the encounter with strangers or acquaintances, who serve as the access points to abstract systems. This encounter is one focus of commentators using the discursive resource of shared decision–making, whereby treatment is understood as a joint venture between patient and healthcare professional:

A37: If something is being tried like this then it’s probably best that the Dr discusses it with the patient, in the same way they would if offering a new drug / therapy as part of a clinical trial. Would probably get better feedback that way.

C7: Not sure about placebos as I’ve never had any, as far as I know but agree that a good doctor can make an enormous difference to a patient and their illness just by listen and discussing the problem in a manner the patient can understand.

Developing this notion, some commentators explicitly took the position of patient as contributor, focussing on the importance of trust, for example, noting:

A38: I told my doctor and my hospital quite clearly and respectfully that I trust their judgement and want to be involved in my care, and that I always insist on knowing what the treatment or medicine is, why it is being given, the side-effects, risks, etc., and that I expect those questions to be answered honestly. I think everyone should be as involved in their care as possible.

One commentator noted that placebo treatment could be conducted ‘in a perfectly honest way, telling patients that this is a treatment that is not proved to work better than a placebo, but can be expected to be as good, and that Placebos are known to work’ (A40). This view reflects a trend in placebo studies research focussed on ‘open-label’ placebo treatment, purportedly bypassing the issue of deception (Carvalho et al., 2016; Colloca and Howick, 2018; Kaptchuk et al., 2010; Kaptchuk and Miller, 2018; Sandler et al., 2010).

The use of shared decision-making as a discursive resource highlights the importance of trust in the therapeutic encounter for placebo treatment, whereby ‘encounters with the representatives of abstract systems … take on the characteristics of trustworthiness associated with friendship and intimacy’ (Giddens, 1990: 85). And, moreover, it offers a way towards understanding placebo treatment as a powerful, productive force bringing patients and healthcare professionals together within a wider communitarian discourse.

Through the notion of the therapeutic encounter, we can situate placebo treatment in a modern conception of evidence-based medicine that promotes expert judgement, shared decision-making and a strong patient–clinician relationship (Charles et al., 1997; Greenhalgh et al., 2014, 2015; Little et al., 2001; Stewart, 2005). This suggests that placebo treatment might sit more comfortably than previously thought within modern medical practice.

## Discussion

In our discursive exploration of public perspectives on placebos and their effects, we highlight two discursive constructs of the placebo: the placebo pill and the treatment process. The consequence of the first, more dominant, construct is an understanding of placebo treatment, informed by an individualist discourse, which divides healthcare professionals and patients. This division, and the paradoxical nature of conceiving of placebos in such a way, closes down potential therapeutic placebo treatment by rendering it illogical and deceptive.

Within the second, counter-discursive construct, we posit that placebo treatment, informed by a communitarian discourse, can be conceptualised as a collective and productive force. We suggest the placebo-as-treatment-process construct can be supported by conceiving of the ‘power of the placebo’ through the therapeutic encounter. Healthcare professionals can promote this notion by taking opportunities to engage patients using the counter-discursive resources of self-healing and shared decision-making. This might open up a productive and collaborative mode of clinical practice by which healthcare professionals could ethically and effectively use placebo treatment. However, although conceiving of placebos in this way seems more intelligible, stretching the definition of placebo treatment so far does raise serious concerns for the utility of the placebo concept itself.

As we have indicated, a modern scientific understanding of placebos and their effects has moved from the ‘psychological’ effects of ‘inert’ substances (Beecher, 1955) towards accounts grounded in different theoretical backgrounds. These include the enculturated meaning of treatment (Brody, 1997; Moerman, 2002); healing rituals and symbols (Kaptchuk and Miller, 2015); motor-intentionality (Frenkel, 2008); embodiment (Thompson et al., 2009) and enactivism (Ongaro and Ward, 2017). However, although these modern theories provide interesting accounts of healthcare practices in general, it is difficult to see how they effectively delineate placebo from non-placebo. All treatment processes can be conceived of in these terms and, therefore, they make no credible argument as to why the placebo is a special case. As Ongaro and Ward (2017: 529) themselves noted, ‘the utility of enactivism for understanding placebo effects stems from its general features as a paradigm for understanding cognition’. Given the competing and confusing discourses that underpin the understanding of placebo treatment, these theories do not offer convincing arguments for why we should talk about placebos in the first place. If we merely want to talk about the potential benefits of the therapeutic encounter, why invoke the confusing and often paradoxical placebo in the first place?

This point has been made previously (Moerman, 2013; Nunn, 2009a, 2009b; Turner, 2012). In response, Howick (2017) developed Grünbaum’s (1986) notion of characteristic and incidental treatment factors as a viable way to delineate non-placebo from placebo. In this model, placebos are treatment processes remedial for a particular disorder, relative to the patient, condition and the therapeutic theory in question (Howick, 2017). For example, the characteristic factor of giving fluoxetine for depression is purportedly the increase of serotonin in the brain through inhibiting reuptake. An incidental factor might be, for example, expectations about receiving the drug. In this way, one can identify the difference between placebo and non-placebo and make a case for the promotion of placebo treatment per se.

However, although the characteristic/incidental distinction makes linguistic sense, questions remain as to its practical use. In line with the findings from this study, a recent review showed that despite an increasingly more coherent understanding of placebos in the research community, both healthcare professionals and patients still broadly conceive of placebos as inert substances (Hardman et al., 2018). If most patients think placebos are ‘inert’ pills having a ‘psychological’ effect, is it just too difficult to convince them otherwise?

Furthermore, is it even clinically useful to group together such a disparate group of factors under the banner of ‘placebo effects’? Some researchers may argue that other perspectives can offer a better way to understand the psychological, social and cultural effects of treatment the placebo concept purports to explain. For example, the Habermasian lifeworld/medical system distinction (Scambler, 2015; Scambler and Britten, 2001), narrative medicine (Charon, 2006) or, as previously noted, enactive or embodied accounts of healthcare practices.

These questions are beyond the scope of this study, but they illustrate that the placebo phenomenon raises important questions about modern evidence-based medicine; about healing; and about the limits of narrow biomedical approaches to healthcare. In addition, whatever one thinks of the clinical viability of placebo treatment, the ‘placebo effect’ still seems an interesting paradigm in which to consider these important medical issues.

### Strengths and limitations of the study

One of the strengths of this study is that by focussing on mainstream media articles related to placebos, we were able to incorporate a broad range of views. However, there are limitations to this approach. In sampling mainstream media, there is a risk that marginalised voices are ignored – it is important that future research on public perspectives on placebos also includes, if possible, a wider range of media and other interactional settings. Moreover, the nature of the data – short comments – may preclude more nuanced reflection by commentators, and this limitation must be considered when reflecting on the findings.

## Conclusion

Based on our discursive exploration of public perspectives on placebos and their effects, we suggest that the dominant way in which placebos are constructed – as inert pills – renders placebo treatment illogical and deceptive. We also identified a counter-discursive construct – the treatment process – through which placebo treatment is grounded in the therapeutic encounter. We suggest that through this construct, placebo treatment might be productively promoted as part of modern evidence-based medicine. However, we also question the clinical worth of stretching the concept of placebo treatment so far, given the current dominant lay understanding. We therefore suggest other theoretical models through which the power of the therapeutic encounter might also be exploited.

In doing so, however, we do not necessarily propose that researchers should stop investigating the placebo phenomenon per se. Merely that by grounding the concept more explicitly in the therapeutic encounter, placebo research would be more orientated to the particular settings in which these ‘placebo effects’ occur. In this sense, we echo recent calls for more ethnomethodological or ethnographic fieldwork in the domain of placebo research (e.g. Hardman et al., 2018; Hutchinson and Moerman, 2018). Such a re-orientation towards ‘the endogenous methods employed by members of societies in the co-production of the order and meaning of clinical settings’ (Hutchinson and Moerman, 2018: 377) may lead to a more fruitful relationship between placebo research and clinical practice.
